# Dr. Ramadge and St. John Long

**Published:** 1831

**Authors:** 


### XXII.

### DR. RAMADGE AND ST. JOHN LONG.

THE letter which has been published with the signature F. H. RAMADGE—FELLOW of the ROYAL COLLEGE of PHYSICIANS, and addressed to St. John Long, is calculated to call forth the most painful reflections in the mind of every man who has the honour of the medical profession at heart. Of the author we know little—and never wish to know more. It is with the LETTER—the *litera scripta* that we have to do, as the legitimate object of criticism. A more disingenuous and sickening epistle never emanated from the press of this or of any other enlightened country. If the LETTER of an individual were capable of disgracing a whole profession, the letter in question is calculated most effectually to do so! While it labours, by every art, and by every species of misrepresentation, to support an ignorant quack, it endeavours, by the same means, to traduce a liberal profession! The wealth of Golconda's mines and the proffer of universal dominion would not induce us to be the author of such a production. Of the objects or intentions of the writer we profess not to judge. They are only known to himself and to his God. But we conceive that the inevitable effect of that letter will be a reprobation of the writer by the whole body of his brethren. It is impossible it can be otherwise. The man who justifies and even extols an ignorant Charlatan, reviling and defaming the regular members of the Faculty at the same moment, cannot expect any thing but indignant contempt from those whom he labours to injure and degrade.*

* Since writing the above, Dr. R. has been expelled the London Medical Society.

This long epistle is thrown into numerical sections, like the counts of an indictment—and no doubt they have *counted* well, both for the inditer and *indicted*. In the *first* count, the writer, of course, professes rigid *impartiality* between the quack and the faculty! He has taken a strange method of shewing his neutrality! In the *second* count there is abundance of hypocrisy, but a lamentable lack of candour. The writer was at first strongly persuaded that Long's treatment of Miss Cashin and Mrs. Lloyd had led to their untimely death. This persuasion was, of course, founded on the evidence of facts. But after a long time—" after deep reflection"—(nothing else?) says the impartial letter-writer, " my opinion is *now* entirely changed." The *third* count tells the quack what the quack would fain forget, namely, that Miss Catherine Cashin was his patient, and that, " apprehending her *disorder* (what disorder?) might become as *precarious* as that which *promised* speedily to terminate in the demise of her sister," he employed precautionary measures, &c. The writer then justifies Long's employment of one or two remedies for all diseases, on the plea that " many eminent practitioners of the present day, are in the habit of employing only one or two remedies in the cure of almost every disease." The tendency of this last assertion, published by a Fellow of the College of Physicians, in a common newspaper, is obvious enough. The assertion itself is as mischievous as it is void of foundation. The writer appears to assume it as a fact, that Miss C. Cashin *did* labour under *disorder* at the time when the quack began his operations—if he had weighed the authentic evidence on this occasion —if he had trusted to facts, and not to fictions, he would have been perfectly satisfied that the lady was in fair and good health.

The *fourth* count is as neat a piece of puffery for Long, as ever emanated from Day and Martin's factory in Holborn. The writer, it seems, has been at immense pains to ascertain, by " a multitude of inquiries," the innocuous qualities of Long's liniment—and in no case—(not even excepting those of Miss Cashin and Mrs. Lloyd?) did the liniment or the inhalation produce the least " noxious or unpleasant effect."

But the *fifth* count is the most uncandid and unprofessional representation that we have ever read.

" The *post-mortem* examination of Miss Cashin satisfactorily proves to me the correctness of your judgment as to the existence of *pulmonary disease*, and which, in my opinion, fully justified you in the steps you took, in the hope of suspending or removing an affection of such fatal tendency."

Will it be believed, that in our own day, a Fellow of the Royal College of Physicians would, in the face of facts recorded on oath by several medical men, come forward and tell the public that the examination of Miss Cashin's body exhibited *pulmonary disease*, to the amount, or to any thing like the amount, which is stated above? This monstrous assertion, too, is made by a man who did not see the body examined, but who coolly avers that the examinators saw what all declare upon oath that they did *not* see, viz. disease of dangerous tendency! This, in fact, is giving the *lie direct* to all the medical witnesses. The writer of the letter, therefore, cannot expect that men, whose veracity is thus unceremoniously impugned, or their evidence distorted, can shew much delicacy, *in manner*, to him who thus insults them.

After the foregoing specimen of veracity and impartiality in the Letter, it is almost an insult to our readers to take further notice of it. The whole is a tissue of presumption, illiberality and special pleading. The following reflection on his brethren is deserving of notice, because we happen to be able to illustrate it.

" As ten days elapsed before Mr. Vance was called in, after Mrs. Lloyd had been under the care of another practitioner, I consider it needless to make any further comment upon his evidence, with this exception, that '*if* you stated Mrs. Lloyd had suffered disease of the chest, which dissection is said not to have proved to be the case,' your mistake is not solitary, for I hardly know *of one* of the profession, of whom I have not heard, and of many of whom I have not *been a personal witness of their erroneous judgments*."

Now for a comment on the above. It is not many months since the writer of this article was requested to visit a young surgeon, lately returned from India, (Mr. Wittle) and then lodging in Tottenham Court-road, in consequence of Dr. Ramadge's decision that there was empyema of the left side of the chest, requiring *paracentis thoracis*. The brother of the patient insisted on Dr. —— seeing him before the operation should be performed. Dr. —— examined the chest, and could discover no sign whatever of empyema. Dr. Barry then visited the patient, and, after careful auscultation and percussion, declared there was not a drop of fluid in the cavity of the pleura! It is hardly necessary to add that the gentleman soon got well without any operation—is now married, and gone to India! So much for Dr. Ramadge's skill in auscultation, and for the numerous errors of judgment which he has witnessed among his brethren. This anecdote, which Dr. —— never before mentioned, either publicly or privately, but which is now called for, on account of the illiberal attack on his brethren, may teach Dr. Ramadge, that he who lives in a house of glass should not be too anxious to get into the habit of throwing stones, lest some panes in his own sky-light be broken in the diversion.

---

